# Improving Biocompatibility of Polyurethanes Apply in Medicine Using Oxygen Plasma and Its Negative Effect on Increased Bacterial Adhesion

**DOI:** 10.1155/2024/5102603

**Published:** 2024-02-23

**Authors:** Kamil Drożdż, Monika Gołda-Cępa, Paulina Chytrosz-Wróbel, Andrzej Kotarba, Monika Brzychczy-Włoch

**Affiliations:** ^1^Department of Molecular Medical Microbiology, Chair of Microbiology, Faculty of Medicine, Jagiellonian University Medical College, Krakow 31-121, Poland; ^2^Faculty of Chemistry, Jagiellonian University, Krakow 31-007, Poland

## Abstract

Polyurethanes (PUs) are versatile polymers used in medical applications due to their high flexibility and fatigue resistance. PUs are widely used for synthetic blood vessels, wound dressings, cannulas, and urinary and cardiovascular catheters. Many scientific reports indicate that surface wettability is crucial for biocompatibility and bacterial adhesion. The use of oxygen plasma to modify PUs is advantageous because of its effectiveness in introducing oxygen-containing functional groups, thereby altering surface wettability. The purpose of this study was to investigate the effect of the modification of the oxygen plasma of polyurethane on its biocompatibility with lung tissue (A549 cell line) and the adhesion of Gram-positive bacteria (*S. aureus* and *S. epidermidis*). The results showed that the modification of polyurethane by oxygen plasma allowed the introduction of functional groups containing oxygen (-OH and -COOH), which significantly increased its hydrophilicity (change from 105° ± 2° to 9° ± 2°) of PUs. Surface analysis by atomic force microscopy (AFM) showed changes in PU topography (change in maximum height from ∼110.3 nm to ∼32.1 nm). Moreover, biocompatibility studies on A549 cells showed that on the PU-modified surface, the cells exhibited altered morphology (increases in cell surface area and length, and thus reduced circularity) without concomitant effects on cell viability. However, serial dilution and plate count and microscopic methods confirmed that plasma modification significantly increased the adhesion of *S. aureus* and *S. epidermidis* bacteria. This study indicate the important role of surface hydrophilicity in biocompatibility and bacterial adhesion, which is important in the design of new medical biomaterials.

## 1. Introduction

Polyurethanes (PUs) are a large family of polymers that are linked by the presence of urethane bonds along molecular chains. The urethane bonds are formed by reacting isocyanates (-NCO) with alcohols (-OH) and consist of small fragments throughout the chains, with macroglycol being the main contributor to these bonds [1]. Medical-grade polyurethane polymers can be found under various brand names, including Carbothane™, Tecoflex™, Tecophilic™, Isoplast® ETPU, Pellethane®, Tecoplast™, Tecothane™, Tecobax™ offered by Lubrizol (USA) [1, 2] and Bionate®, Bionate® II, CarboSil®, Biomerix, Elasthane®, ATPU, Biospan® by DSM (the Netherlands) [1, 3]. PUs are used in the manufacture of catheters, heart valves, vascular grafts, prostheses [1, 4], pacemakers [5], dialysis devices, wound dressings, and breast implant shells [6]. They owe such widespread use in medicine to their high flexibility and fatigue resistance [6]. The biological response correlates very strongly with the surface properties of biomaterials [1]. Cell adhesion to synthetic surfaces is influenced by the type of functional groups, surface charge, topography, and also wettability [7]. It is believed that the wettability of the surface of biomaterials is the most important parameter affecting biocompatibility [7, 8]. It is the wettability of the surface that controls the adhesion of cellular proteins and thus affects the cellular response. Studies report that cells adhere best to surfaces with a wetting angle of 40° to 70° [9, 10]. On the other hand, however, there are studies that suggest that the more hydrophilic the surface, the better and faster cell adhesion [11, 12]. One way to increase the biocompatibility of the surface of polymeric materials is to incorporate oxygen-containing groups. Studies indicate that low-temperature plasma modification is effective in modifying polyurethanes [13]. The method is often used due to its high functionalisation efficiency and ease of implementation. In addition, its use is in line with the principles of green chemistry (zero-waste process). Plasma interaction with polymeric materials can be divided into three stages: surface cleaning (e.g., removal of organic contaminants), chemical modification (e.g., breaking C-C and C=C bonds and forming functional groups), and surface etching and nanotopography formation (consequences of prolonged exposure) [14].

Despite extensive biocompatibility studies, an important aspect of the development of new biomaterials is their susceptibility to microbial adhesion, which leads to the occurrence of infections and related complications. When bacteria adhere to the surface of medical devices and multiply in a suitable environment, a biofilm develops, in which bacteria are well protected from antibiotics and immune system factors [15]. According to the literature, 80% of implant infections are most often caused by staphylococci, of which *Staphylococcus aureus* and *Staphylococcus epidermidis* account for about 75% of these infections [16]. The purpose of this study was to investigate the effect of oxygen plasma modification of polyurethane on its biocompatibility with lung tissue (A549 cell line) and the adhesion of Gram-positive bacteria (*S. aureus* and *S. epidermidis)*.

## 2. Materials and Methods

### 2.1. Preparation and Functionalisation of the Material

The film, made of medical-grade polyurethane with a thickness of 100 *μ*m, was purchased from American Polyfilm, Inc. Before functionalisation, the polyurethane films were cut to the size of a 14 mm diameter disc, then rinsed with 2-propanol (Avantor), and allowed to air dry. Surface modification of the polyurethanes was carried out by treating them with oxygen plasma (FEMTO system, Diener Electronics) at a controlled partial pressure of oxygen of 0.14 mbar (Oxygen SIAD Poland 6.0 purity 99.9999%), plasma generator power of 50 W, and sample exposure time of 5 minutes.

### 2.2. X-Ray Photoelectron Spectroscopy

The surface composition was checked by XPS in an ultrahigh vacuum system equipped with an SES R4000 analyzer (Gammadata Scienta). A monochromatic Al K*α* source (1486.6 eV) operating at 350 W was used. The spectra were obtained at a take-off angle of 90°. The vacuum in the spectrometer chambers was better than 5 × 10^−9^ mbar. The obtained XPS spectra were analysed using Casa-XPS 2.3.15 software. All spectra were calibrated using a random C 1s peak with a fixed value of 285 eV. The surface concentrations of polyurethane components were determined by integrating narrow scans of C 1s and O 1s maxima.

### 2.3. Contact Angle

The wettability of unmodified and low-temperature oxygen plasma-modified surfaces was measured using a Surftens Universal instrument (OEG GmbH) in a thermal chamber. Static water contact angles were calculated using Surftens 4.3 on the Windows platform. Three independent water droplets of 2.5 *μ*l were used in each experiment. The average value was calculated over five independent measurements.

### 2.4. Atomic Force Microscopy and Image Processing

To characterize the topography of unmodified and low-temperature oxygen plasma-modified surfaces, atomic force microscopy in contact mode was used. Measurements were performed with a JPK NanoWizard 4 XP system equipped with an Olympus optical microscope and an active vibration isolation platform (Accurion i4). All the experiments were conducted under air and ambient conditions in an acoustic enclosure. A Bruker scanning probe, model TESPG-V2 equipped with an antimony (Sb) doped silicon (Si) tip, was utilized. Topographic data were processed using JPK Data Processing 7.0.162 software from a 1 *μ*m^2^.

### 2.5. Cell Line Cultures

Biocompatibility and viability tests were performed on the human A549 ATCC cell line (CRL-185™ American Type Culture Collection), derived from lung tissue. Cells were cultured in DMEM (Gibco) supplemented with 10% fetal bovine serum (Gibco) in culture bottles (Nest) at 37°C and 5% CO atmosphere. ZellShield antibiotic kit® (Minerva Biolabs) was also added to the medium.

### 2.6. Live/Dead Cell Line Staining with FDA and PI

The tested polyurethane surfaces were sterilised with UV light for 20 minutes and pressed against the bottom of the plate with a sterile quartz ring. Fluorescein diacetate (FDA) (Sigma) and propidium iodide (PI) (Sigma) were used to assess cell viability. For live-dead assays, A549 cells were seeded in a 1 ml volume containing 3 × 10^4^ cells in a 24-well plate (Nest). Inoculation of the biomaterial surface was carried out for 24 hours. After this time, the culture fluid was removed, and the cells were incubated for 10 min with 1 ml of 1x concentrated phosphate buffer (PBS, Chempur®, pH = 7.4) containing 0.8 *μ*l of FDA (5 mg/ml) and 25 *μ*l of PI (2 mg/ml). After this time, the biomaterials were washed with PBS and transferred to a primary slide and sealed with a coverslip in a drop of PBS. Stained cells were visualised on an Olympus BX63 fluorescence microscope. Images were taken on the FITC and TRITC channels, which were used for green (live cells) and red (dead cells) fluorescence, respectively. The experiments were performed in triplicates.

### 2.7. Cytoskeleton Staining

Phalloidin conjugated with green dye and DAPI were used to evaluate the biocompatibility of the tested surfaces. For this purpose, the A549 canine lineage (CRL-185™ ATCC) was seeded in 1 ml containing 3 × 10^4^ cells in a 24-well plate (Nest). Polyurethane surfaces were sterilised with UV light for 20 min and pressed with a sterile quartz ring. Inoculation of the biomaterial surfaces lasted 24 h. After this time, the cells were fixed with 4% paraformaldehyde in PBS (Sigma) for 20 minutes, washed with PBS, permeabilized in 0.5% Triton X-100 (Sigma), and diluted in PBS. The biomaterials were then washed again with PBS. Nucleic acid was stained with DAPI (NucBlueTM Fixed Cells Stain, Invitrogen™), while the cytoskeleton (F-actin fibers) was stained with AlexaFluor™ 488 Phalloidin (ActinGreen™ 488 ReadyProbes™ Reagent, Invitrogen) according to the manufacturer's protocol. Biomaterials were thoroughly washed with PBS, transferred to a primary slide, and sealed with a coverslip in a drop of PBS. The images of stained cells were recorded using an Olympus BX63 fluorescence microscope. Images were taken on the DAPI and FITC channels, which were used for blue (stained DNA) and green (stained F-actin) fluorescence, respectively. The images were analysed with the use of Fiji Image J 1.53 software. The following parameters were quantified: the area, length (major axis), and circularity of the cells. Cell circularity was calculated according to the formula 4*π∗area*/*perimeter*^2^ which equals 1.0 for perfect circle shape. All the experiments were performed in triplicate.

### 2.8. Adhesion of Bacteria

Two Gram-positive strains of *S. aureus* DSM (Deutsche Sammlung von Mikroorganismen und Zellkulturen) 4910 and *S. epidermidis* DSM 28319 were used in the study. All isolates were placed in a Microbank™ system (Pro-Lab Diagnostics Inc.) and stored at −20°C for further analysis. Strains were thawed by seeding on agarose medium supplemented with 5% sheep blood (Becton Dickinson) and incubated overnight at 37°C. For the experiment, a bacterial colony was suspended in 10 ml of tryptic soy broth (TSB) (Becton Dickinson) and incubated for 18 h at 37°C.

The experiment was conducted in two systems. The first was an 18-hour culture in tryptic soy broth (TSB), where the bacterial cells are coated with various molecules present in the medium. The second study system was conducted in PBS, in which the native surface charge of the bacteria is exposed. To expose the bacterial surface charge, the 18 h culture was washed 3 times in PBS by centrifugation at 3.000 RPM for 4 min.

Static bacterial adhesion was conducted in 24-well plates (Nest), UV light sterilised, and pressed to the bottom with the plate with sterile quartz rings. Each biomaterial was incubated for 30 min, 60 min, and 240 min with 0.5 ml of a 10^6^ CFU/ml bacterial suspension. Afterwards, the discs were gently washed 3 times with PBS to remove unattached bacterial cells from the surface.

The number of bacteria on the surface of biomaterials was assessed using the serial dilution method. For this purpose, 0.25% trypsin (BD Difco™) diluted in PBS was used and bacteria were mechanically washed from the surface of the polyurethanes. Then, serial 10-fold dilutions of the resulting bacterial suspension were made, and 100 *μ*l was seeded on tryptic soybean agar (TSA) medium (Dickinson) for each dilution in duplicate. Plates were incubated for 24 h at 37°C. After this time, the bacterial colonies were counted, and the results are given in colony-forming unit (CFU) per ml.

The area occupied by bacteria on the polyurethane surface was also evaluated. For this purpose, an Olympus BX63 fluorescence microscope with UPlanXApo20x/0.80 objective was used. The bacteria were stained with SYTO9 and PI stains included in the LIVE/DEAD BacLight Bacterial Viability Kit (Life Technologies) according to the manufacturer's recommendations. The area occupied by bacteria (%) was assessed using the Fiji Image J 1.53 software by summing the bacteria on the FITC channel (green channel-live bacteria) and the TRITC channel (red channel-dead bacteria) based on 30 photos taken on the tested surfaces. All experiments were conducted in triplicate.

### 2.9. Statistical Analysis

Statistical analysis was conducted using the IBM SPSS Statistics 28 package. The normality of the distribution of continuous variables was checked using the Shapiro–Wilk test. To compare the mean values of biocompatibility parameters, Student's t-test was employed. In the case of assessing adhesion to the tested surfaces, a multivariate analysis of variance (ANOVA) model was used in scheme 3 (incubation time: 30 vs. 60 vs. 240 minutes) × 2 (medium: PBS vs TSB) × 2 (surface: unmodified vs modified) along with a Bonferroni post hoc test. Results were considered statistically significant when *p* < 0.05.

## 3. Results and Discussion

### 3.1. X-Ray Photoelectron Spectroscopy

**Figure 1:**
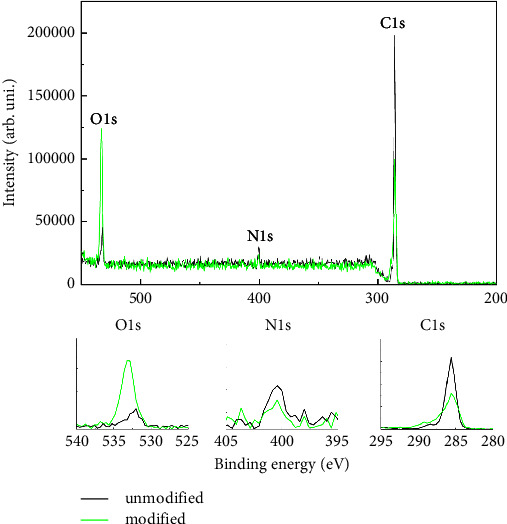
XPS results for unmodified (black) and plasma-treated (green) polyurethane surfaces.

X-ray photoelectron spectroscopy (XPS) was used to confirm the presence of oxygen-containing functional groups introduced onto polyurethane surfaces using low-temperature oxygen plasma. The XPS survey spectra and subsequent narrow scan analysis for electrons C, O, and N have provided valuable insights into the composition and chemical states of the analysed samples. In the survey spectra, three major peaks were prominently detected. O 1s is at 533.2 eV, C 1s is at 285.6 eV, and N 1s is at 400.4 eV. To gain a deeper understanding of the chemical environment of these elements, narrow scans were performed. The narrow scan analysis revealed additional subpeaks and chemical shifts associated with specific bonding configurations and oxidation states for each element, i.e., changes in the O 1s peak from 532.0 eV to 533.2 eV. N 1s at a binding energy of 400.4 eV correspond to the isocyanate groups (-N-C(=O)-) in urethane links [17]. These findings summarize the main differences in the XPS spectra after plasma modification of polyurethane, which are a decrease in the intensity of the C 1s peak and a significant increase in the O 1s peak. This indicates the introduction of oxygen onto the polyurethane surface (see Figure 1). These changes were achieved using low oxygen pressure at 0.2 mbar and a brief functionalisation duration of 5 minutes. The analysis of tensile strength and Young's modulus values for polyurethane films before and after plasma treatment confirms that the mild plasma conditions used do not significantly affect their mechanical properties [18].

### 3.2. Wetting Angle

**Figure 2:**
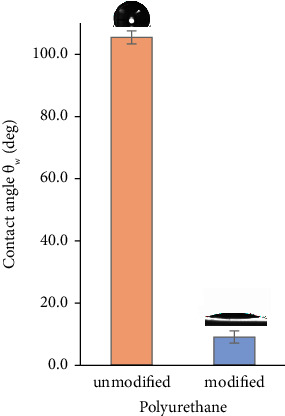
Contact angle values (*θ*_*W*_) for unmodified and plasma-treated polyurethane samples. Representative pictures of the water droplets on the tested surfaces are presented above the bars.

The XPS results confirmed the introduction of oxygen-containing functional groups; based on our previous reports, these groups are mostly -OH and -COOH [19]. Their introduction affects the surface wettability, which can be measured by means of contact angle measurements. The unmodified polyurethane samples are hydrophobic, having a contact angle *θ*_*W*_ = 105° ± 2°. After exposure to plasma, the surface becomes hydrophilic, which is evident in the reduction of the contact angle value to *θ*_*W*_ = 9° ± 2 (see Figure 2). A superhydrophilic surface is called such a material whose wetting angle is less than 5° [20]. In our study, a surface close to superhydrophilic was obtained. In addition, the low value of the standard deviation shows the high reproducibility of the polyurethane surface modification. The application of oxygen plasma is a widely recognized method for enhancing the wettability of PU materials [21, 22].

### 3.3. Atomic Force Microscopy

**Figure 3:**
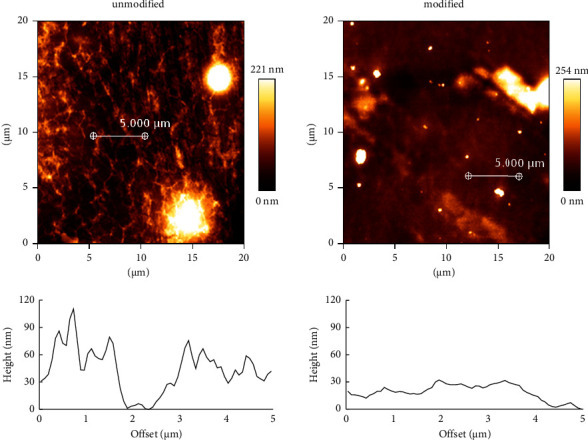
AFM surface topography of unmodified and modified polyurethane with low-temperature oxygen plasma (FEMTO system, Diener Electronics) for 5 min, 0.14 mbar, and 50 W. The cross sections include graphs showing the surface height profile before and after low-temperature oxygen plasma treatment at 5 *μ*m for 5 min.

Plasma modification affects not only the surface chemistry but also the topography as a result of material etching [14]. To visualize the changes in surface roughness and topography, AFM observations were performed. The images collected from the area of 20 *μ*m × 20 *μ*m are presented in Figure 3. The unmodified polyurethane has an irregular topography. Enormous differences in height can be observed in the cross-section diagram (below the image of the AFM images). After the polyurethane is exposed to the plasma, the surface is smoothened. The maximum altitude decreased from ∼110.3 nm to ∼32.1 nm. Changes in the topography of polymers may be caused by etching the polyurethane or removing impurities from its surface after the polymer film production process.

Surface etching of polyurethanes is a frequently observed phenomenon during their plasma modification [14]. However, too long exposure times can lead to undesirable structural damage of the coating [23] or degradation [24], especially if thin polymeric films are being modified. Therefore, it is important to limit the modification to the top layer of the polymer by fine optimization of plasma modification parameters.

### 3.4. Live/Dead Cell Line Staining with FDA and PI

**Figure 4:**
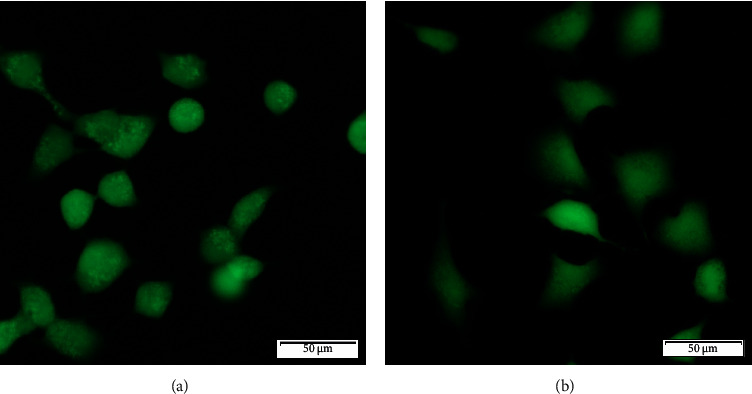
FDA/PI staining of the A549 cell line (CRL-185™ ATCC) on unmodified (a) and modified polyurethane (b) using oxygen plasma (FEMTO system, Diener Electronics) for 5 min, 0.14 mbar, and 50 W.

The results of the A549 cells viability (CRL-185TM ATCC) were performed using FDA and PI stains showing that the modified and unmodified biomaterial did not adversely affect the cell line. Cells showed remarkably high viability on both tested surfaces as indicated by the high percentage of viable (>95%) (green) cells on the tested surfaces (see Figure 4). Scientific reports indicate that polyurethanes are nontoxic surfaces [1]. These results confirm the lack of toxicity of polyurethanes and the absence of negative effects of introduced functional groups on PU surfaces. Similar results were obtained by Vlaisavljevich et al., who incubated fibroblasts for 24 h and 48 h with polyurethane coatings obtaining viability >98% [25]. Liu et al. also showed viability >90% on thermoplastic polyurethanes after 24 h and 48 h incubation of HaCaT and NIH3T3 lines [26]. Moreover, Stevenson Jr. et al. tested catheters made of polyurethane, which also showed no cytotoxic effect against HUVEC after 72 h [27].

### 3.5. Cytoskeleton Staining

Biocompatibility is a complex phenomenon and depends on a variety of elements. Thanks to it, host cells tolerate artificial implants without triggering unwanted reactions from the immune system, such as allergic reactions and inflammation, and moreover, the process of tumorigenesis is not affected. These properties must be met in the short or long term, depending on the purpose of the polymer. In long-term applications, a bioactive surface is additionally required, that is, a surface that will allow host cell adhesion. However, the surface of polymeric materials, including polyurethanes, is known for its inertness. For this reason, it is necessary to modify the surface to increase bioactivity [28].

**Figure 5:**
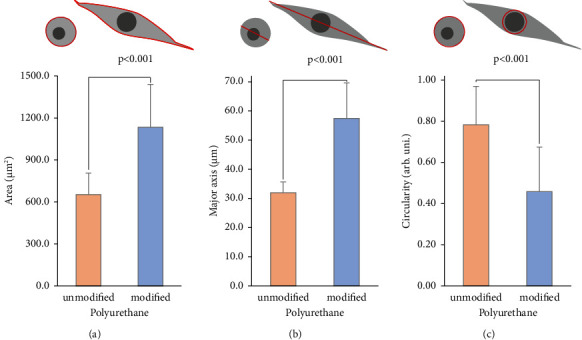
Evaluation of the morphology shape of the A549 (CRL-185™ ATCC) cell line after 24 h incubation on the tested polyurethane surfaces. (a) Average cell surface area, (b) average cell length, and (c) average cell circularity.

**Figure 6:**
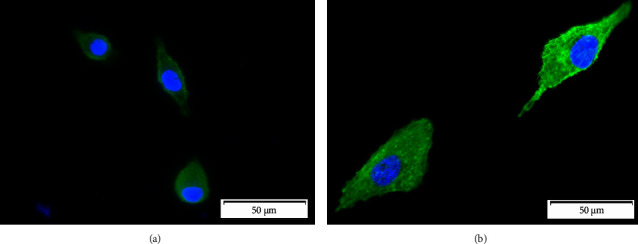
Cell line morphology of A549 (CRL-185™ ATCC) with F-actin labelled (green, ActinGreen™ 488 ReadyProbes™ reagent, Invitrogen™) and cell nucleus (blue, NucBlue™ fixed cell stain, Invitrogen™) on unmodified polyurethane (a) and modified oxygen plasma (b) (FEMTO system, Diener Electronics) for 5 min, 0.14 mbar, and 50 W.

The effect of oxygen plasma modification of the polyurethane surface significantly affected the adhesion of the A549 cell line (CRL-185™ ATCC). Cells on the modified surface had a significantly larger surface area (an average increase of 480.0 *μ*m^2^, see Figure 5(a)) and greater length of the major axis (an average increase of 25.5 *μ*m, see Figure 5(b)). However, the cell circularity coefficient decreased significantly (average decrease of 0.3, see Figure 5(c)). Changes in cell morphology were also confirmed by microscopic imaging (see Figure 6).

An important property affecting biocompatibility is surface wettability. It plays a key role in the interaction between proteins and thus host cells. It has been noted that hydrophilic surfaces bind proteins better and are more biocompatible than hydrophobic surfaces [7, 29]. On the oxygen plasma-modified surfaces, the cells had a bipolar or tripolar morphology. This is indicated by the parallel orientation of actin filaments with a long cell axis [30]. The results obtained for the above study also confirm that hydrophilic surfaces (such as modified PU) show significantly higher biocompatibility compared to hydrophobic surfaces (e.g., unmodified PU). This was proven by an increase in cell size and length of the major axis of symmetry and a decrease in circularity on polyurethane treated with low-temperature oxygen plasma. Identical dependence of fibroblastic morphology of NIH3T3 cell line was shown on hydrophilic (*θ*_*W*_ = 5°) graphene surfaces functionalized with oxygen plasma [31]. Moreover, as indicated by other research works, the presence of oxygen-containing functional groups on the surface of biomaterials stimulates cell adhesion and proliferation [32, 33]. In contrast, on hydrophobic surfaces, cell adhesion is low, and cells remain rounded and show a low rate of proliferation [34]. All the results presented so far were obtained during in vitro experiments, so extrapolation of these results to an in vivo model should be done with due caution.

#### 3.5.1. Evaluation of Adhesion of Bacteria in PBS and TSB to Polyurethane Surfaces by Serial Dilution and Plate Count

Regardless of the species assessed and the system used (incubation in TSB or PBS), a significant increase in bacteria was observed on the surface of modified polyurethane compared to unmodified samples (*p* < 0.05).

**Figure 7:**
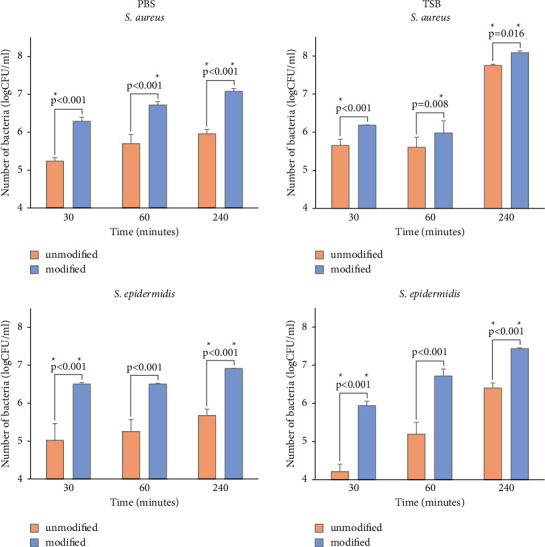
Evaluation of the adhesion of the bacteria *S. aureus* DSM 4910 and *S. epidermidis* DSM 28319 to unmodified and modified polyurethane using the serial dilution method. Adhesion was assessed in PBS buffer (smooth charts) and TSB growth medium (dot pattern) after 30, 60, and 240 mins of incubation. Statistically significant differences between the unmodified and modified surfaces are marked with lines. Statistically significant differences between PBS and TSB between the same surface types are marked ^*∗*^ (*p* < 0.05). Error bars stand for the standard deviation.

When bacterial adhesion was conducted in PBS, it can be clearly seen that after 30 minutes of incubation, more *S. aureus* bacterial cells adhered to the modified surface than to the unmodified polyurethane. In addition, on the modified surface, the number of bacteria grew faster at successive measurement points (60 and 240 minutes), while on the unmodified surface, a less dynamic increase in the number of bacteria was observed. On the other hand, when bacterial adhesion was conducted in TSB, the difference in the number of bacteria on the surfaces tested after 30 minutes of incubation was smaller. An additional 30 minutes of incubation did not result in a greater number of attached *S. aureus* on modified and unmodified surfaces. A significant increase was observed after 240 minutes (see Figure 7).

Analogous trends in adhesion to the tested surfaces in PBS were observed in bacteria of the species *S. epidermidis*. After 30 minutes of incubation, the number of bacteria on the modified surface is significantly higher than on the unmodified polyurethane surface. The difference in the number of bacteria on the modified and unmodified surface persisted until the end of 240 minutes. However, when assessing the adhesion of *S. epidermidis* bacteria in TSB, we observed a pattern different from that of *S. aureus*. In the case of *S. aureus*, there was no significant increase in bacterial numbers after 30 and 60 minutes of incubation on both the unmodified and modified surfaces. On the contrary, *S. epidermidis* showed sustained bacterial growth at all time points (30, 60, and 240 minutes) on both study surfaces. The results obtained for the adhesion of *S. epidermidis* in TSB show similar trends as for the adhesion of this species in PBS to the tested surfaces (see Figure 7).

#### 3.5.2. Evaluation of Adhesion of Bacteria in PBS and TSB to Polyurethane Surfaces Using a Fluorescence Microscope

Similarly, to the serial dilution method, regardless of the examined bacterial strains and the experimental methodology used (incubation in TSB or PBS), a notable elevation in the bacterial population was observed on the surface of the modified polyurethane compared to the unmodified control samples (*p* < 0.05).

**Figure 8:**
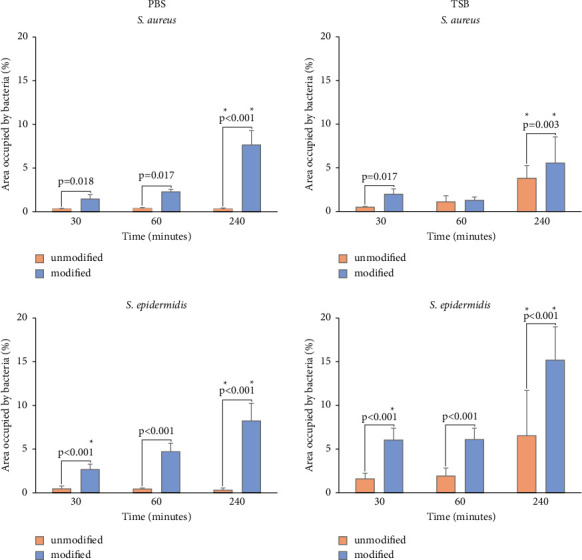
Evaluation of the occupied surface of unmodified and modified polyurethanes by *S. aureus* DSM 4910 and *S. epidermidis* DSM 28319 polyurethanes using a fluorescence microscope (SYTO9/PI). Adhesion was assessed in PBS buffer (smooth charts) and TSB growth medium (dot pattern) after 30, 60, and 240 mins of incubation. Statistically significant differences between the unmodified and modified surfaces are marked with lines while differences between PBS and TSB and the same surface types are marked ^*∗*^ (*p* < 0.05). Error bars stand for the standard deviation.

Bacterial adhesion was also evaluated by fluorescence microscopy using SYTO9 and PI fluorescent dyes. Incubation of *S. aureus* in PBS with the tested surfaces showed that the modification introduced on the polyurethane significantly increased the number of bacteria on its surface. The difference between the unmodified and modified surfaces in the percentage of the area occupied by bacteria after 60 minutes of incubation did not significantly increase, and a significant increase was observed after 240 minutes of incubation for *S. aureus* on the modified surfaces. If *S. aureus* adhesion was carried out in TSB, then a greater difference was observed after 30 minutes of incubation between the modified and unmodified surfaces in the percentage of the field occupied by *S. aureus* on the tested surfaces. This difference after an additional 30 minutes of incubation was decreased close to zero, but after 240 minutes of incubation, greater bacterial adhesion to the modified polyurethane surfaces could be observed again (see Figure 8).

Similar patterns in adhesion to the surfaces evaluated in PBS were observed in *S. epidermidis* bacteria as in the serial dilution method. After 30 minutes of incubation, the percentage of occupied area by bacteria on the modified surfaces was significantly higher than on the unmodified polyurethane surface. Again, the modified surface exhibited superior adhesion compared to the unmodified surface, and this persisted until the 240 minutes of incubation. The same trend in bacterial adhesion as in PBS was observed for *S. epidermidis* adhesion when the experiment was conducted in TSB. After 30 minutes of incubation, there were more bacteria on the modified surfaces than on the unmodified, and moreover, the differences were similar to those in the experiment with PBS. The difference in the number of bacteria on the tested surfaces continued up to 240 minutes of incubation time (see Figure 8).

The attachment of bacteria to non-living surfaces is significantly influenced by surface characteristics (chemical composition and/or morphology). The initial adhesion of bacteria to abiotic surfaces is mediated by nonspecific forces (Lifshitz-van der Waals, Lewis acid-base forces, and electrostatic forces) [35]. However, it is crucial to recognize that hydrophobic bacterial cells exhibit greater adhesion to hydrophobic surfaces, whereas cells with a hydrophilic nature strongly adhere to hydrophilic surfaces. It is worth noting, however, that the bacterial population does not have a fixed hydrophilic or hydrophobic phenotype. Microorganisms can switch between hydrophobic and hydrophilic phenotypes in response to changes in environmental conditions and growth stages [36]. In our study, two species of the genus Staphylococcus were used: *S. aureus* and *S. epidermidis*. When superhydrophobic surfaces were produced on polyurethane sponges with zinc oxide and copper nanoparticles [37] or polyurethane-coated titanium [38], *S. aureus* adhesion decreased when compared to control samples. In contrast, in our case, bacterial adhesion to hydrophilic surfaces of both *S. aureus* and *S. epidermidis* was significantly increased. Bacterial adhesion is also affected by surface topography. In general, an increase in surface roughness promotes bacterial adhesion [39]. In addition, microorganisms tend to preferentially occupy cavities present on the surface of biomaterials [40]. The large surface area to which bacteria can attach increases the number of contact points between the material surface and bacterial cells, which result in increased adhesion [39, 40]. In our case, plasma etching resulted in a smoother surface; however, it did not reduce the number of bacteria. Therefore, based on our results, it seems that the mechanism of interaction related to surface wettability and nonspecific electrostatic forces is more important than the surface morphology.

## 4. Conclusions

In the present study, the effect of oxygen functional groups introduced on polyurethane surfaces using low-temperature oxygen plasma on the biocompatibility of the A549 cell line, and the adhesion of two Gram-positive bacterial species (*S. aureus* and *S. epidermidis*) was evaluated. The modification changes the surface chemistry by increasing its hydrophilicity and smoothing its topography. The results of this study provide information on the role of surface hydrophilicity in biocompatibility and bacterial adhesion to surfaces. The introduction of oxygen functional groups significantly influenced biological aspects such as biocompatibility but, at the same time, increased bacterial adhesion. Therefore, when designing new medically relevant materials, in addition to biocompatibility assessment, attention should also be paid to bacterial adhesion.

## Data Availability

The data supporting the current study are available from the corresponding author upon request.

## References

[1] Wendels S., Avérous L. (2021). Biobased polyurethanes for biomedical applications. *Bioactive Materials*.

[2] Lubrizol (2024). *Medical Polymers*.

[3] DSM Biomedical (2024). *Medical Polyurethanes*.

[4] Sobczak M., Kędra K. (2022). Biomedical polyurethanes for anti-cancer drug delivery systems: a brief, comprehensive Review. *International Journal of Molecular Sciences*.

[5] Navas-Gómez K., Valero M. F. (2020). Why polyurethanes have been used in the manufacture and design of cardiovascular devices: a systematic Review. *Materials*.

[6] Rusu L. C., Ardelean L. C., Jitariu A.-A., Miu C. A., Streian C. G. (2020). An insight into the structural diversity and clinical applicability of polyurethanes in biomedicine. *Polymers*.

[7] Jurak M., Wiącek A. E., Ładniak A., Przykaza K., Szafran K. (2021). What affects the biocompatibility of polymers?. *Advances in Colloid and Interface Science*.

[8] Zimina A., Senatov F., Choudhary R. (2020). Biocompatibility and physico-chemical properties of highly porous PLA/HA scaffolds for bone reconstruction. *Polymers*.

[9] Al-Azzam N., Alazzam A. (2022). Micropatterning of cells via adjusting surface wettability using plasma treatment and graphene oxide deposition. *PLoS One*.

[10] Arima Y., Iwata H. (2007). Effect of wettability and surface functional groups on protein adsorption and cell adhesion using well-defined mixed self-assembled monolayers. *Biomaterials*.

[11] Jo W.-L., Lim Y.-W., Kwon S.-Y. (2023). Non-thermal atmospheric pressure plasma treatment increases hydrophilicity and promotes cell growth on titanium alloys in vitro. *Scientific Reports*.

[12] Wei J., Yoshinari M., Takemoto S. (2007). Adhesion of mouse fibroblasts on hexamethyldisiloxane surfaces with wide range of wettability. *Journal of Biomedical Materials Research Part B: Applied Biomaterials*.

[13] Duch J., Golda-Cepa M., Kotarba A. (2019). Evaluating the effect of oxygen groups attached to the surface of graphenic sheets on bacteria adhesion: the role of the electronic factor. *Applied Surface Science*.

[14] Abusrafa A., Habib S., Popelka A. (2020). Surface functionalization of a polyurethane surface via radio-frequency cold plasma treatment using different gases. *Coatings*.

[15] Singh S., Singh S. K., Chowdhury I., Singh R. (2017). Understanding the mechanism of bacterial biofilms resistance to antimicrobial agents. *The Open Microbiology Journal*.

[16] Oliveira W. F., Silva P. M. S., Silva R. C. S. (2018). Staphylococcus aureus and Staphylococcus epidermidis infections on implants. *Journal of Hospital Infection*.

[17] Baskakov S. A., Baskakova Y. V., Kabachkov E. N. (2023). On the state of graphene oxide nanosheet in a polyurethane matrix. *Nanomaterials*.

[18] Chytrosz-Wrobel P., Golda-Cepa M., Stodolak-Zych E., Rysz J., Kotarba A. (2023). Effect of oxygen plasma-treatment on surface functional groups, wettability, and nanotopography features of medically relevant polymers with various crystallinities. *Applied Surface Science Advances*.

[19] Chytrosz-Wrobel P., Golda-Cepa M., Drozdz K. (2023). In vitro and in silico studies of functionalized polyurethane surfaces toward understanding biologically relevant interactions. *ACS Biomaterials Science and Engineering*.

[20] Wang Z., Paul S., Stein L. H., Salemi A., Mitra S. (2022). Recent developments in blood-compatible superhydrophobic surfaces. *Polymers*.

[21] West J. O. F., Critchlow G. W., Lake D. R., Banks R. (2016). Development of a superhydrophobic polyurethane-based coating from a two-step plasma-fluoroalkyl silane treatment. *International Journal of Adhesion and Adhesives*.

[22] Ghorbani F., Zamanian A. (2018). Oxygen-plasma treatment-induced surface engineering of biomimetic polyurethane nanofibrous scaffolds for gelatin-heparin immobilization. *E-polymers*.

[23] Bauer M. G., Reithmeir R., Lutz T. M., Lieleg O. (2021). Wetting behavior and stability of surface-modified polyurethane materials. *Plasma Processes and Polymers*.

[24] Hegemann D., Brunner H., Oehr C. (2003). Plasma treatment of polymers for surface and adhesion improvement. *Nuclear Instruments and Methods in Physics Research Section B: Beam Interactions with Materials and Atoms*.

[25] Vlaisavljevich E., Janka L. P., Ong K. G., Rajachar R. M. (2011). Magnetoelastic materials as novel bioactive coatings for the control of cell adhesion. *IEEE Transactions on Biomedical Engineering*.

[26] Liu M., Liu T., Chen X. (2018). Nano-silver-incorporated biomimetic polydopamine coating on a thermoplastic polyurethane porous nanocomposite as an efficient antibacterial wound dressing. *Journal of Nanobiotechnology*.

[27] Stevenson A. T., Reese L. M., Hill T. K. (2015). Fabrication and characterization of medical grade polyurethane composite catheters for near-infrared imaging. *Biomaterials*.

[28] Cvrček L., Horáková M. (2019). Plasma modified polymeric materials for implant applications. *Non-Thermal Plasma Technology for Polymeric Materials*.

[29] Xu L.-C., Siedlecki C. A. (2007). Effects of surface wettability and contact time on protein adhesion to biomaterial surfaces. *Biomaterials*.

[30] Golda-Cepa M., Chorylek A., Chytrosz P. (2016). Multifunctional PLGA/parylene C coating for implant materials: an integral approach for biointerface optimization. *ACS Applied Materials and Interfaces*.

[31] Golda-Cepa M., Kumar D., Bialoruski M. (2023). Functionalization of graphenic surfaces by oxygen plasma toward enhanced wettability and cell adhesion: experiments corroborated by molecular modelling. *Journal of Materials Chemistry B*.

[32] Gentleman M. M., Gentleman E. (2014). The role of surface free energy in osteoblast–biomaterial interactions. *International Materials Reviews*.

[33] Recek N. (2019). Biocompatibility of plasma-treated polymeric implants. *Materials*.

[34] Golda-Cepa M., Engvall K., Hakkarainen M., Kotarba A. (2020). Recent progress on parylene C polymer for biomedical applications: a Review. *Progress in Organic Coatings*.

[35] Arciola C. R., Campoccia D., Montanaro L. (2018). Implant infections: adhesion, biofilm formation and immune evasion. *Nature Reviews Microbiology*.

[36] Krasowska A., Sigler K. (2014). How microorganisms use hydrophobicity and what does this mean for human needs?. *Frontiers in Cellular and Infection Microbiology*.

[37] Ozkan E., Mondal A., Singha P. (2020). Fabrication of bacteria- and blood-repellent superhydrophobic polyurethane sponge materials. *ACS Applied Materials and Interfaces*.

[38] El-Chami, Mikhael F., Jane Mayotte (2020). Reduced bacterial adhesion with parylene coating: potential implications for micra transcatheter pacemakers. *Journal of Cardiovascular Electrophysiology*.

[39] Song F., Koo H., Ren D. (2015). Effects of material properties on bacterial adhesion and biofilm formation. *Journal of Dental Research*.

[40] Zheng S., Bawazir M., Dhall A. (2021). Implication of surface properties, bacterial motility, and hydrodynamic conditions on bacterial surface sensing and their initial adhesion. *Frontiers in Bioengineering and Biotechnology*.

